# Preoperative Transient Elastography in Patients with Esophageal Cancer

**DOI:** 10.3390/diagnostics12123194

**Published:** 2022-12-16

**Authors:** Tzu-Yi Yang, Chia-Pang Shih, Pei-Ching Huang, Chun-Yi Tsai, Yin-Kai Chao

**Affiliations:** 1 Division of Thoracic Surgery, Chang Gung Memorial Hospital, Linkou Branch, Taoyuan City 333, Taiwan; 2 Department of Nursing, Yuanpei University of Medical Technology, Hsin-Chu City 300, Taiwan; 3 Department of Medical Imaging and Intervention, Chang Gung Memorial Hospital, Linkou Branch, Taoyuan City 333, Taiwan; 4 Department of General Surgery, Chang Gung Memorial Hospital, Linkou Branch, Taoyuan City 333, Taiwan

**Keywords:** esophageal cancer, alcoholic cirrhosis, hepatic failure, esophagectomy, transient elastography, indocyanine green, postoperative complications

## Abstract

Since excessive alcohol consumption is a shared risk factor for esophageal cancer and liver fibrosis, it is possible that patients with esophageal cancer may develop unknown liver fibrosis or cirrhosis. We applied preoperative transient elastography (TE) to patients without recorded cirrhosis undergoing esophagectomy to clarify the validity in predicting postesophagectomy hepatic failure. The cohort consisted of 107 patients who received TE before esophagectomy between June 2018 and December 2021. Patients were categorized into two groups based on the fibrosis score yielded by preoperative TE (mild group: 0~2, n = 92; severe group: 3~4, n = 15). There was no significant difference in demographic data nor surgical variables between the two groups. None of the cohort encountered hepatic failure, yet the severe fibrosis group had a significantly higher rate of pleural effusion (40.0% versus 15.2%, *p* = 0.03). The areas under the curve (AUCs) of TE in predicting postoperative complications and 180-day mortality were 0.60 (95% CI: 0.46–0.74) and 0.67 (95% CI: 0.51–0.83), respectively. In conclusion, stratification of patients with esophageal cancer who had liver fibrosis by preoperative TE demonstrates significant validity in predicting postoperative pleural effusions. Recruitment of noncirrhotic patients with higher TE scores is warranted to examine its power in other parameters.

## 1. Introduction

Esophageal cancer is the eighth most common cancer and the sixth leading cause of cancer death worldwide [1]. In addition to the devastating prognosis at an advanced stage, the rising incidence of esophageal cancer also highlights the need for early detection for prompt treatment [2]. Excessive alcohol consumption was considered one of the risk factors for esophageal cancer [3]. It is also an established contributing factor for developing liver cirrhosis, which accounts for 7% of patients with esophageal cancer [4]. In patients with concomitant esophageal cancer and liver cirrhosis, the morbidity and mortality of esophagectomy rise from 33% to 87% and 3% to 26%, respectively [5,6]. Furthermore, postoperative hepatic failure leads to up to 75% of mortality after esophagectomy for patients with cirrhosis, even though they did not undergo hepatectomy [7]. Based on this scenario, preoperative evaluation of liver fibrosis should raise surgeons’ concerns before performing radical esophagectomy for esophageal cancer patients with a history of excessive alcohol consumption and the absence of liver cirrhosis.

There are omnifarious laboratory tests and images to demonstrate hepatic insufficiency and to achieve the diagnosis of liver cirrhosis. For patients with hepatocellular carcinoma (HCC) who will undergo hepatectomy, the indocyanine green (ICG) clearance test was applied to determine the feasibility and extent of hepatectomy and to predict posthepatectomy hepatic failure [8]. Oda et al. applied the ICG test to exclude esophagectomy for patients with a potential risk of postoperative hepatic insufficiency or failure [9]. However, we still encounter patients who developed postesophagectomy hepatic failure under the strict selection criteria. For patients with mild to moderate fibrosis, evaluation with noninvasive tests is common and practical [10]. Transient elastography (TE), a noninvasive, painless, rapid and easy-to-perform examination, is considered a valid diagnostic tool for liver fibrosis and cirrhosis [11]. In this study, we applied TE as a preoperative evaluation for patients without remarkable liver cirrhosis who will undergo esophagectomy in order to clarify the validity in predicting postesophagectomy hepatic insufficiency or failure.

## 2. Material and Methods

### 2.1. Study Design and Patient Inclusion

**Figure 1:**
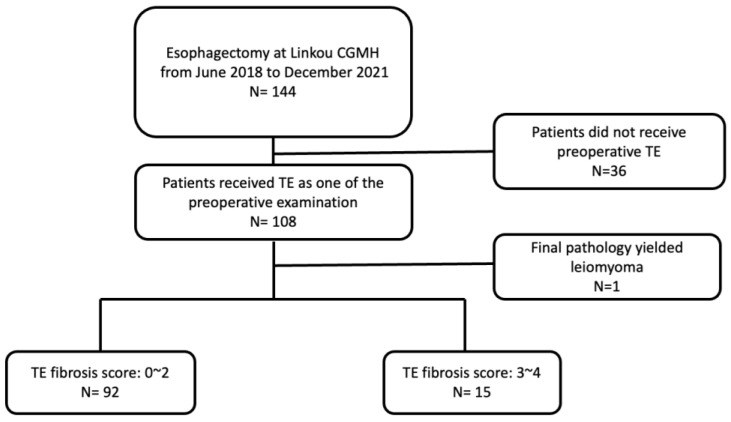
Algorithm of patient inclusion for the current study.

This is a prospectively designed, retrospectively registered, single-institute study based in Chang Gung Memorial Hospital, Linkou. Figure 1 depicts the algorithm of patient selection throughout the study. Patients with esophageal cancer who underwent thoracic esophagectomy and reconstruction with gastric conduits between June 2018 and December 2021 were enrolled. Patients who did not receive TE within one month prior to esophagectomy were excluded. One case whose final pathology report was leiomyosarcoma was excluded. The cohort, consisting of 107 patients, was categorized into two groups based on the fibrosis score yielded by the preoperative TE. The primary endpoint of this study was postesophagectomy hepatic insufficiency or failure. The postoperative complications, which were stratified based on the Clavien–Dindo classification [12], were also analyzed in order to demonstrate the potential correlation with different extents of liver fibrosis.

### 2.2. Surgical Procedures

All patients underwent minimally invasive esophagectomy in the McKeown fashion. Fifty-six patients (52%) underwent a robotic-assisted approach. The harvest of the gastric conduit was performed via a totally laparoscopic approach, except for two patients who underwent conversion to laparotomy owing to severe intraperitoneal adhesion resulting from the previous operations. Gastric pull-up was performed mostly through the retrosternal route (n = 94, 87%), which was created laparoscopically after the harvest phase of the gastric conduit. All of the esophagogastrostomy anastomoses were performed at the level of the cervical esophagus via a left lateral neck incision.

### 2.3. Transient Elastography

TE was performed by a single hepatologist within one month prior to the operation. Measurements were performed on the right lobe of the liver through the intercostal spaces while the patient was in the dorsal decubitus position with the right arm in maximal abduction. The probe transducer was placed on the skin and located a liver portion of at least 6 cm thick, free of large vascular structures. Ten successful acquisitions were performed on each patient. The median value was taken as the liver elastic modulus, and the liver stiffness measurement (LSM) was expressed in kilopascal (kPa). The values were shifted from LSM to fibrosis scores of Metavir based on stratification of alcoholic liver disease [13]. The cut-off values for scores 1, 2, 3, and 4 were set at 5.9 kPa, 7.5 kPa, and 9.5 kPa, respectively. Scores equal to or above 3 were classified as having significant fibrosis.

### 2.4. Perioperative Outcomes

Short-term outcome measures of postoperative complications included pneumonia, pleural effusion and anastomosis leakage. Pneumonia was defined according to the Revised Uniform Pneumonia Score [14]. The other two complications, pleural effusion and anastomosis leakage, were defined according to the system proposed by the Esophagectomy Complications Consensus Group [15]. Patients who were intubated again after successful extubation for 12 h were recorded as reintubated. Readmission to the intensive care unit (ICU) was recorded if patients were transferred back to the ICU from an ordinary ward.

### 2.5. Statistical Analysis

The normality of the distribution of numerical variables was tested with the Shapiro–Wilk test. We categorized patients into mild and severe groups by fibrosis score, as well as ICG test results. Categorical variables were expressed as absolute values and percentages, whereas continuous variables were presented as the mean and standard deviation or median and (q1, q3). We used the chi-square test or Fisher’s exact test to compare categorical variables between study groups. We conducted a two-sample t test or Mann–Whitney U test to compare differences in numerical variables. To analyze liver enzyme changes before and after the operation, the Generalized Estimating Equations (GEEs) were used. SAS version 9.4 (SAS Inc., Cary, NC, USA) was used for analysis and p values >.05 were considered statistically significant.

## 3. Results

### 3.1. Patient Characteristics and Surgical Parameters

Ninety-two patients in the cohort (86%) who had TE scores 0~2 were stratified into the mild group, whereas the remaining 15 patients (14%) with TE scores 3~4 were in the severe group. Table 1 demonstrates the characteristics of the two groups. Except for the preoperative serum ALT level, there was no significant difference in age, tumor location, preoperative clinical cancer staging, postoperative serum AST level or bilirubin level between the two groups. Patients in the mild fibrosis group had significantly higher levels of ALT than those in the severe fibrosis group (median (q1, q3): 27.0 (18.5, 43.0) versus 19.0 (13.0, 27.0), *p* = 0.02). Regarding the parameters related to surgery, which included operation time, intraoperative blood loss, the rate of conversion to laparotomy during the harvest and the routes for gastric pull-up, there was no significant difference between the two groups (Table 2).

### 3.2. Perioperative Outcomes

Regarding the primary endpoint of this study, none of the patients suffered from postoperative hepatic failure. Except for the significantly higher rate of postoperative pleural effusions in the severe fibrosis group than in the mild fibrosis group (40.0% versus 15.2%, *p* = 0.03), there was no significant difference in the rate of postoperative reintubation, rates of postoperative readmission to the ICU, rates of postoperative pneumonia, leakage of the anastomosis or the presence of ≥grade III postoperative complications between the two groups (Table 3). Nevertheless, there was no difference in the median postoperative hospital stay between the two groups (13 days versus 14 days, *p* = 0.42). Only one patient in the mild fibrosis group died because of nosocomial pneumonia after the operation.

Patients who had preoperative ICG clearance tests within the cohort (N = 75) were categorized into two groups by the cutoff ICG clearance at 10%. The identical perioperative parameters were analyzed again and the results are shown in Table 4. However, none of these parameters revealed significant differences between the two groups.

### 3.3. The Validity to Predict the Rate of Postoperative Complications and 180-Day Mortality

**Figure 2:**
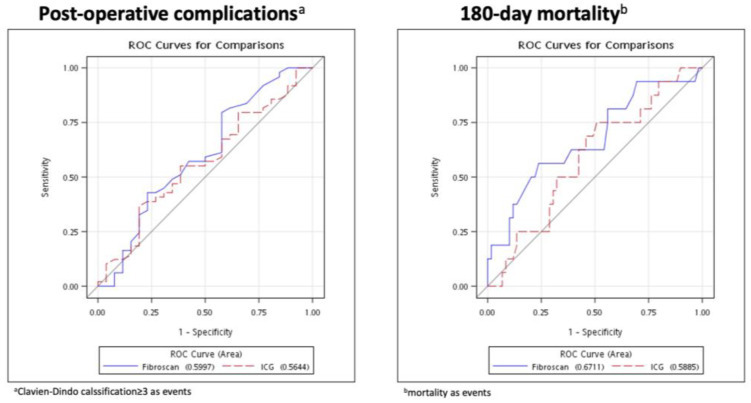
ROC curves of postoperative complications and 180-day mortality with comparison between TE and ICG test. Abbreviations: TE, transient elastography; ICG, indocyanine green.

Figure 2 depicts the receiver operating characteristic curve (ROC) of postoperative complications (Clavien–Dindo classification ≥ 3) and the 180-day mortality of the TE in comparison to the ICG test. In postoperative complications, the area under the curve (AUC) of the ROC of the TE and the ICG tests were 0.60 (95% CI: 0.46–0.74) and 0.56 (95% CI: 0.43–0.70), respectively. On the other hand, the AUCs of the ROC curves in predicting 180-day mortality of the TE and the ICG tests were 0.67 (95% CI: 0.51–0.83) and 0.59 (95% CI: 0.44–0.74), respectively.

### 3.4. The Fluctuation of Liver Enzymes during the Perioperative Period

**Figure 3:**
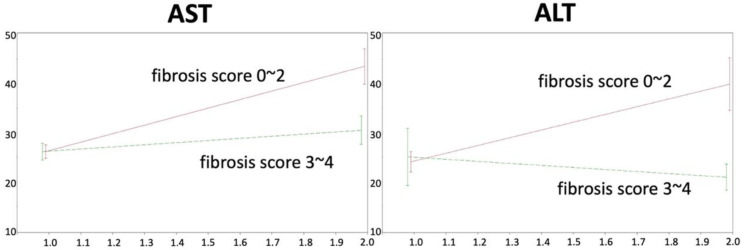
GEE analysis of liver enzymes before and after esophagectomy. Abbreviations: AST, aspartate transaminase; ALT, alanine acetyltransferase.

Although there was no postesophagectomy hepatic failure in our cohort, we recorded the fluctuation of liver enzymes during the perioperative period and analyzed the results with GEE in order to demonstrate the subtle damage to the liver. Figure 3 illustrates the fluctuation of AST and ALT between the two groups. GEE models were utilized to compare the changes in AST and ALT before and after the operation in the two groups. The change in AST before and after the operation was significantly lower in the severe fibrosis group than in the mild fibrosis group (β = −0.4; *p* = 0.003) after adjusting for age, sex and cirrhosis. Similarly, the change in ALT before and after the operation was significantly lower in the severe fibrosis group than in the mild fibrosis group (β = −0.6; *p* = 0.006) after adjusting for age, sex and cirrhosis.

## 4. Discussion

Patients with liver cirrhosis had a higher risk of morbidity and mortality after major operations, and esophageal cancer surgery is of no exception. Owing to the higher rates of postoperative complications, Child-Pugh class B and C liver cirrhosis are regarded as one of the contraindications of esophagectomy [16]. In patients with Child-Pugh class A liver cirrhosis, Trivin et al. reported that there was no difference in prognosis between these patients and noncirrhotic patients [17]. However, Cheng et al. concluded that esophagectomy portended a higher rate of postoperative morbidities in patients with Child-Pugh class A liver cirrhosis [7]. Since alcohol consumption is a shared risk factor for developing esophageal cancer and alcoholic cirrhosis, we believe that patients diagnosed with esophageal cancer carry different extents of liver injury or even fibrosis after excessive exposure to alcohol. All the above findings implied that patients with esophageal cancer eligible for esophagectomy were evaluated and potentially selected without remarkable liver fibrosis or hepatic insufficiency. However, we still encounter patients suffering from postesophagectomy hepatic insufficiency after these modalities of selection. The Child-Pugh score is composed of serum albumin level, serum bilirubin level, prothrombin time, the amount of ascites and the presence of hepatic encephalopathy [18]. Nevertheless, the three serologic tests could not completely represent the complicated hepatic functions including catabolism, detoxication, anabolism and bile secretion [19,20,21]. This might partially answer the inconsistency between the low preoperative Child-Pugh score and the occurrence of postesophagectomy hepatic insufficiency or failure. To fill this gap, Oda et al. reported the application of a preoperative ICG clearance test, which represents the hepatic secretory function, to predict the rate of postoperative complications after esophagectomy [9], even though patients did not undergo hepatectomy. 

TE was proposed for decades as a valid tool to quantify liver fibrosis whether it resulted from viral hepatitis, fatty liver or excessive alcohol consumption [11]. Although the cutoff point of the TE results was different, the TE results were proven to be correlated with postoperative outcomes [8,22,23]. However, most of the proposed studies focused on the validity of TE in patients undergoing hepatectomy. Since the ICG clearance test could be applied to evaluate postoperative morbidities and possible hepatic insufficiency on patients undergoing esophagectomy without hepatectomy, we speculated that preoperative TE might also provide relevant information. The execution of the ICG clearance test is composed of serological sampling and quantification at different times (5 min, 10 min and 15 min, respectively) after the single-dose parenteral injection of the ICG. The 15-min clearance was retrieved after linear regression of the three results. This ICG test is more complicated than performing TE. Furthermore, TE is absolutely less invasive than the ICG test. Although the patients in our cohort demonstrated various extents of liver fibrosis based on the preoperative TE, none of the patients died of postesophagectomy hepatic insufficiency during the study period. Nonetheless, patients with higher TE scores did not suffer from a significantly higher rate of postoperative complications, except for the presence of pleural effusion. This result could be explained by our setting of inclusion criteria that the cohort consisted of esophageal cancer patients with asymptomatic liver fibrosis, and probably led to the attenuation of the power of TE in discrimination. This is also a limitation of our study, in that the cohort was designated for esophagectomy based on the so-called “normal” ICG test result. 

Postesophagectomy pleural effusion was the only significant postoperative complication between our two groups stratified by different TE fibrosis scores. Our previous retrospective review of esophageal cancer patients who underwent esophagectomy concluded that patients with Child-Pugh class A cirrhosis had higher rates of postoperative pleural effusion, chylothorax and postoperative pneumonia, as well as longer intensive care unit stays by propensity score matching than all other patients. Since our current cohort is composed of patients with different extents of liver fibrosis, less so than cirrhosis, it is rational that the postoperative complications are less than those of cirrhotic patients. However, our current results demonstrated that stratification by TE could provide discrimination for some postesophagectomy complications in patients with mild liver fibrosis. 

**Figure 4:**
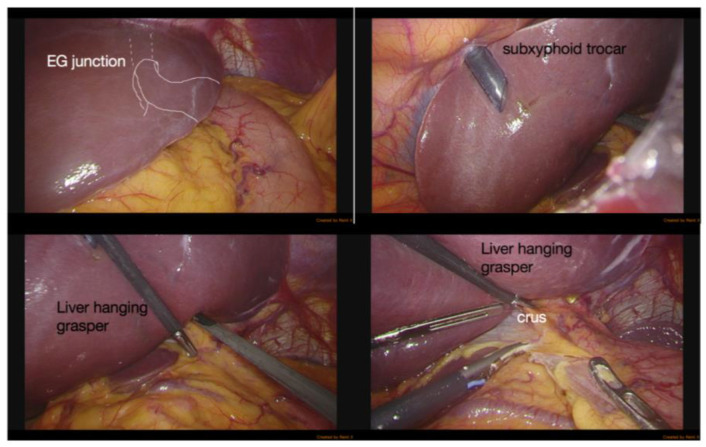
“Sling and expose” maneuver of liver during laparoscopic gastric conduit harvest.

Regarding the GEE of liver enzymes, the subgroup with a TE fibrosis score < 2 demonstrated significant fluctuation compared with the other subgroup, which is contrary to our presumption. Elevation of liver enzymes correlated with damage to the hepatocytes, including physical injuries, such as contusion [24]. Liver retraction during laparoscopic upper gastrointestinal surgery is crucial to expose the field of operation, [25,26] especially those involving the esophagogastric junction (EGJ). Figure 4 depicts the maneuver of liver retraction during harvesting of the gastric conduit by the laparoscopic approach, which was universally performed in our cohort. Based on the GEE results showing that the softer livers presented with relatively significant elevation of liver enzymes, we speculated that softer livers had healthier hepatocytes and were more vulnerable to physical damage. Although this phenomenon is self-limited without significant sequelae, we will probably adjust the liver retraction maneuver to avoid possible damage.

There are several limitations to our study. First, the fibrosis score we applied was based on a meta-analysis focusing on patients with chronic viral hepatitis [13]. There is no current consensus for the cutoff value for alcohol-related fibrosis. Reevaluation of our cohort once the TE results about alcoholic fibrosis have been updated are necessary. Second, our cohort was composed of selected patients who met the criteria of the ICG clearance test. Meanwhile, there were only 12 patients with a fibrosis score 3~4. To clarify the power of TE in evaluating postesophagectomy hepatic insufficiency, more patients with normal ICG but high fibrosis scores measured from TE are warranted in the future.

## 5. Conclusions

Stratification of patients with esophageal cancer who had liver fibrosis fall short of cirrhosis by preoperative TE could predict the presence of postoperative pleural effusion based on our study. However, the same examination did not demonstrate significant differences in postesophagectomy hepatic failure or other related complications. The AUC of TE in predicting postoperative complications and 180-day mortality were similar to the preoperative ICG test. We believe that recruitment of more noncirrhotic patients with higher TE fibrosis scores is warranted to clarify the power of TE in the prediction of postesophagectomy hepatic insufficiency or failure.

## Figures and Tables

**Table 1:** Characteristics of the two groups.

Variables	Mild (n = 92)	Sever (n = 15)	*p*-Value
Age, mean ± std	57.0 ± 8.9	54.3 ± 5.5	0.12 ^b^
Sex, male, n (%)	87 (94.6%)	15 (100%)	1.00 ^c^
Tumor histology, n (%)			1.00 ^c^
SCC	83 (90.2%)	14 (93.3%)	
Adenocarcinoma	9 (9.7%)	1 (6.7%)	
Tumor location, n (%)			0.42 ^c^
Upper third	9 (9.8%)	2 (13.3%)	
Middle third	43 (46.7%)	4 (26.7%)	
Lower third	38 (40.3%)	9 (60.0%)	
ECJ	2 (2.2%)	0 (0.0%)	
Clinical stage ^a^, n (%)			0.60 ^c^
I	7 (7.6%)	2 (13.3%)	
II	10 (10.9%)	1 (6.7%)	
III	59 (63.1%)	8 (53.3%)	
IV	16 (17.4%)	4 (26.7%)	
Preoperative CCRT, n (%)			0.13 ^c^
Yes	79 (85.9%)	10 (66.7%)	
No	13 (14.1%)	5 (33.3%)	
Postoperative laboratory test, median (q1, q3)			
AST (U/L)	36.5 (27.0, 48.0)	28.0 (22.0, 41.0)	0.12 ^d^
ALT (U/L)	27.0 (18.0, 42.0)	19.0 (13.0, 27.0)	0.02 ^d^
Total bilirubin (mg/dL)	0.4 (0.3, 0.6)	0.5 (0.3, 0.6)	0.55 ^d^
Albumin (g/dL)	4.2 (3.9, 4.5)	4.2 (4.0, 4.5)	0.93 ^d^
INR	1.0 (1.0, 1.1)	1.1 (1.0, 1.1)	1.00 ^d^

Mild: Fibrosis score 0~2; Severe: Fibrosis score 3~4; SCC: squamous cell carcinoma; ECJ: esophagocardiac junction; CCRT: concurrent chemoradiotherapy; AST: aspartate transaminase; ALT: alanine acetyltransferase; INR: International normalized ratio. ^a^ According to American Joint Committee on Cancer, 8th edition. ^b^ *p*-value was performed by two-sample t-test. ^c^ *p*-value was performed by Fisher’s exact test. ^d^ *p*-value was performed by Mann–Whitney U test.

**Table 2:** Surgical variables of the two groups.

Variables	Mild (n = 92)	Sever (n = 15)	*p*-Value
Operation time (minutes), median (q1, q3)			
	448.5 (409.5, 511.0)	474.0 (426.0, 510.0)	0.25 ^d^
Blood loss (mL), median (q1, q3)			
	67.5 (50.0, 150.0)	100.0 (70.0, 105.0)	0.32 ^d^
Surgical type of thoracic part, n (%)			1.00 ^c^
VATS	45 (48.9%)	7 (46.7%)	
RATS	47 (51.1%)	8 (53.3%)	
Conversion of abdominal part, yes, n (%)	1 (1.1%)	1 (6.7%)	0.26 ^c^
Thoracic duct ligation, n (%)	71 (77.2%)	11 (73.3%)	0.75 ^c^
Reconstruction route, n (%)			1.00 ^c^
Retrosternal	80 (87.0%)	13 (86.7%)	
Posterior mediastinum	12 (13.0%)	2 (13.3%)	
Pathology stage ^a^, n (%)			0.11 ^c^
I	50 (54.4%)	6 (40.0%)	
II	21 (22.8%)	3 (20.0%)	
III	20 (21.7%)	4 (26.7%)	
IV	1 (1.1%)	2 (13.3%)	

Mild: Fibrosis score 0~2; Severe: Fibrosis score 3~4; VATS: Video-assisted thoracoscopic surgery; RATS: Robotic-assisted thoracoscopic surgery. ^a^ According to American Joint Committee on Cancer, 8th edition. ^c^ *p*-value was performed by Fisher’s exact test. ^d^ *p*-value was performed by Mann–Whitney U test.

**Table 3:** Surgical outcomes of the two groups.

Variables	Mild (n = 92)	Sever (n = 15)	*p*-Value
Reintubation, yes, n (%)	2 (2.2%)	1 (6.7%)	0.36 ^c^
Readmission to ICU, yes, n (%)	6 (6.5%)	2 (13.3%)	0.31 ^c^
Pneumonia, yes, n (%)	9 (9.8%)	2 (13.3%)	0.65 ^c^
Leakage from anastomosis, yes, n (%)	14 (15.2%)	2 (13.3%)	1.00 ^c^
Pleural effusion, yes, n (%)	14 (15.2%)	6 (40.0%)	0.03 ^c^
Major complication ^a^, yes, n (%)	33 (35.9%)	7 (46.7%)	0.57 ^c^
LOS (days), median (q1, q3)	13 (11, 17)	14 (12, 22)	0.42 ^d^
In-hospital mortality, yes, n (%)	1 (1.1%)	0 (0.0%)	1.00 ^c^

ICU: intensive care unit; LOS: length of stay. ^a^ Clavien–Dindo calssification≥3. ^c^ *p*-value was performed by Fisher’s exact test. ^d^ *p*-value was performed by Mann–Whitney U test.

**Table 4:** Surgical outcomes of the two study groups based on ICG test results.

Variables	<10% (n = 57)	≥10% (n = 17)	*p*-Value ^c^
Reintubation, yes, n (%)	2 (3.5%)	0 (0.0%)	1.00
Readmission to ICU, yes, n (%)	4 (7.0%)	0 (0.0%)	0.57
Pneumonia, yes, n (%)	7 (12.3%)	0 (0.0%)	0.19
Leakage from anastomosis, yes, n (%)	8 (14.0%)	2 (11.8%)	1.00
Pleural effusion, yes, n (%)	12 (21.1%)	1 (5.8%)	0.28
Major complication ^a^, yes, n (%)	21 (35.1%)	5 (29.4%)	0.78
In-hospital mortality, yes, n (%)	1 (1.8%)	0 (0.0%)	1.00

^a^ Clavien–Dindo classification ≥3. ^c^ *p*-value was performed by Fisher’s exact test.

## Data Availability

Due to its ethic concern, data related to the study cannot be made openly available. Please refer to the corresponding author C.-Y.T., if access of the data is warranted in any circumstance.
